# Supplementary material for: Uncovering the dynamics and consequences of RNA isoform changes during neuronal differentiation
Source: Mol Syst Biol. 2024 May 16;20(7):3. doi: 10.1038/s44320-024-00039-4 (PMC11219738; doi:10.1038/s44320-024-00039-4)
Supplement: Supplementary file 1 — Appendix [file 44320_2024_39_MOESM1_ESM.pdf]

Uncovering the dynamics and consequences of RNA isoform changes during neuronal differentiation

Contents

Appendix Figure S1 (related to Figure 2)...2
Appendix Figure S2 (related to Figure 4)...3
Appendix Figure S3 (related to Figure EV3)..4
Appendix Table S1 (related to Figure 1)..5

Appendix Figure S1

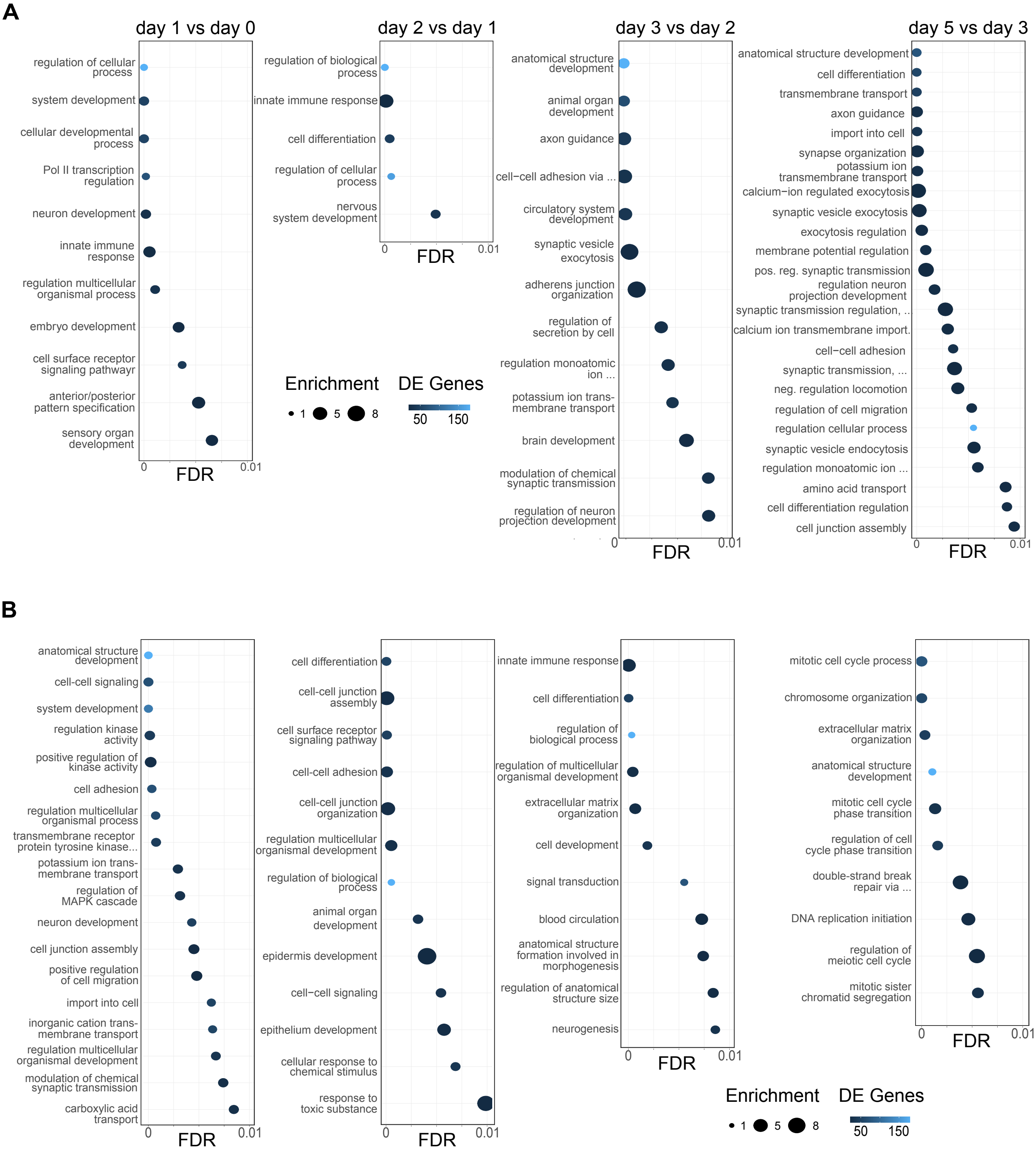

Appendix Figure S1: GO term analysis of the differentially expressed genes during NGN3m differentiation (related to Figure 2). (A, B) Significant GO-slim terms (FDR<0.01, hypergeometric test; level 0) from **(A)** up- and **(B)** down-regulated differentially expressed genes during the differentiation course.

Appendix Figure S2

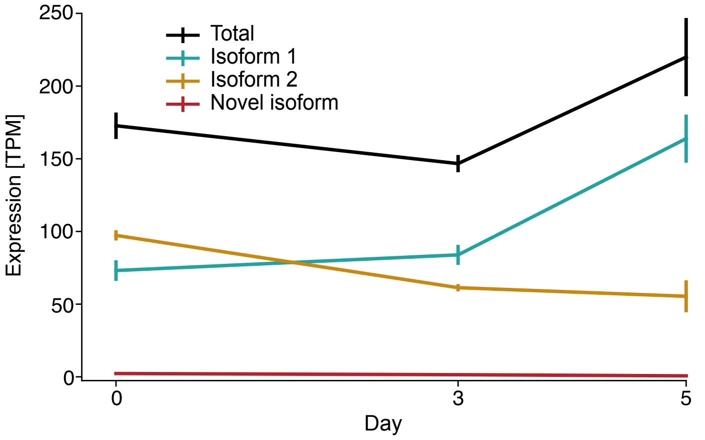

Appendix Figure S2: PFN2 expression during human neuronal cell differentiation (related to Figure 4). Quantification of total gene expression of PFN2 and relative abundance of three transcript isoforms inferred from ONT-seq time-course data. Expression profiles are shown for two annotated isoforms and one a novel transcript isoform. Expression levels were normalized as transcript per million (TPM).

Appendix Figure S3

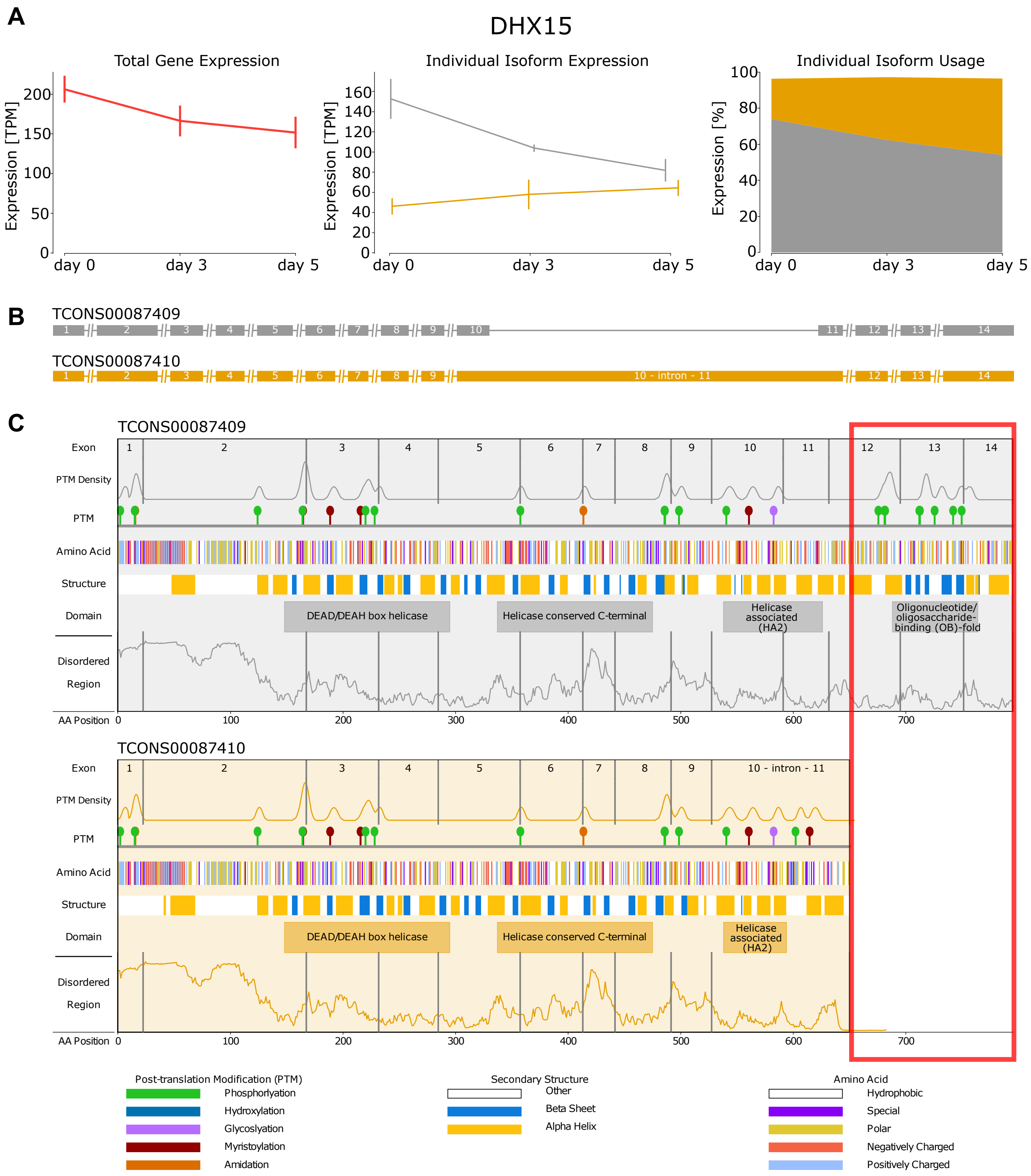

Appendix Figure S3: Visualization of functional features of two DHX15 transcript isoforms and their predicted translated products (related to Figure EV3). (A) Quantification of total gene expression of DHX15 and relative abundance of two transcript isoforms as detected in ONT-seq time-course data. **(B)** Schematic view of the exon-intron structures of two DHX15 transcript isoforms. TCONS00087409 and TCONS00087410 correspond to ENST00000336812.5 and a novel isoform in Figure EV3E, respectively. One retained intron between exon 10 and 11 in TCONS00087410 was detected. **(C)** Visualization of functional annotations of the two DHX15 transcript isoforms and their predicted translated products by IsoTV (Annaldasula et al., 2021). The functional features include post-translational modifications (PTMs), secondary structures, chemical properties of amino acids, and protein domains.

Appendix Table S1

Appendix Table S1: Primers used for the RT-qPCR analysis (related to Figure 1)

Gene	Forward primer	Reverse primer
NANOG	CCTATGCCTGTGATTTGTGGG	TTTGGGACTGGTGGAAGAATC
SOX2	GTACAACTCCATGACCAGCTC	CTTGACCACCGAACCCAT
Oct4	GCAGAAAGAACTCGAGCAATTTG	CAGATGGTCGTTTGGCTGAATAC
NES	GCACCTCAAGATGTCCCT	GTCCTGAAAGCTGAGGGAA
NOTCH1	AGTTGTGCTCCTGAAGAACG	CCATATGATCCGTGATGTCCC
DCX	AGCAGTCTCCCATCTCTACG	TTGGATTTGTACTCTGGACTCTG
MAP2	GAAAAGGCCCAAGCTAAAGTTG	CTGTGTAATGATCTCAGCCCC
TUBB3	CCTCCGTGTAGTGACCCTT	GGCCTTTGGACATCTCTTCAG
ACTB	ACCTTCTACAATGAGCTGCG	CCTGGATAGCAACGTACATGG
B	GTACTCAATCCGGTCCTGC	GGGTCACGCTGAAGTATTCTG
SDE2	ATCAGTGAGAATCGGAAACGG	TCTCAGAGTTGGACCCTTCT
