# Supplementary figures and images for: Uncovering the dynamics and consequences of RNA isoform changes during neuronal differentiation
Source: Mol Syst Biol. 2024 May 16;20(7):3. doi: 10.1038/s44320-024-00039-4 (PMC11219738; doi:10.1038/s44320-024-00039-4)

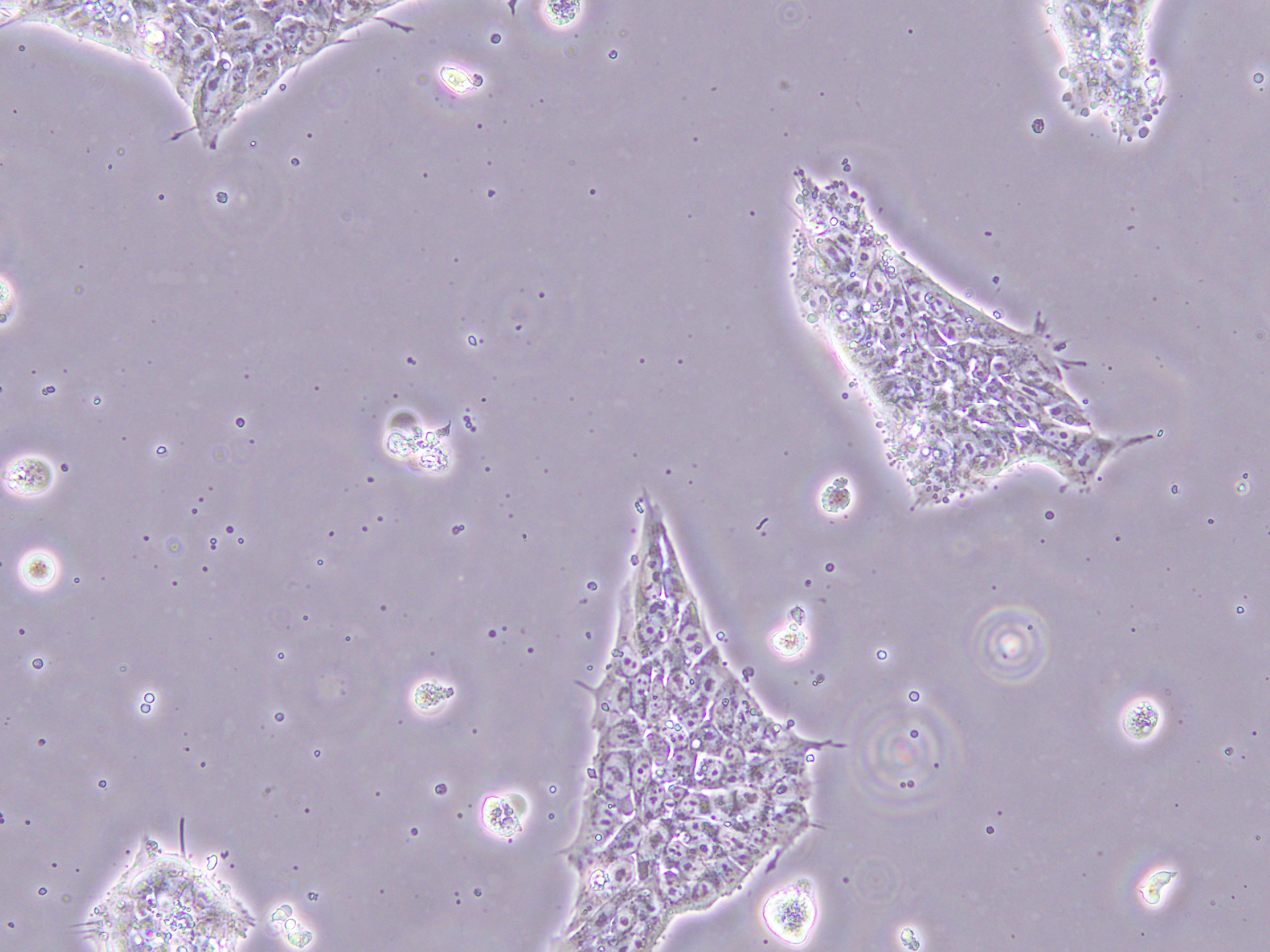

Supplement: Supplementary file 2 — Source data Fig. 1 [file 44320_2024_39_MOESM2_ESM.zip › Figure1/B/D0_20x.tif]

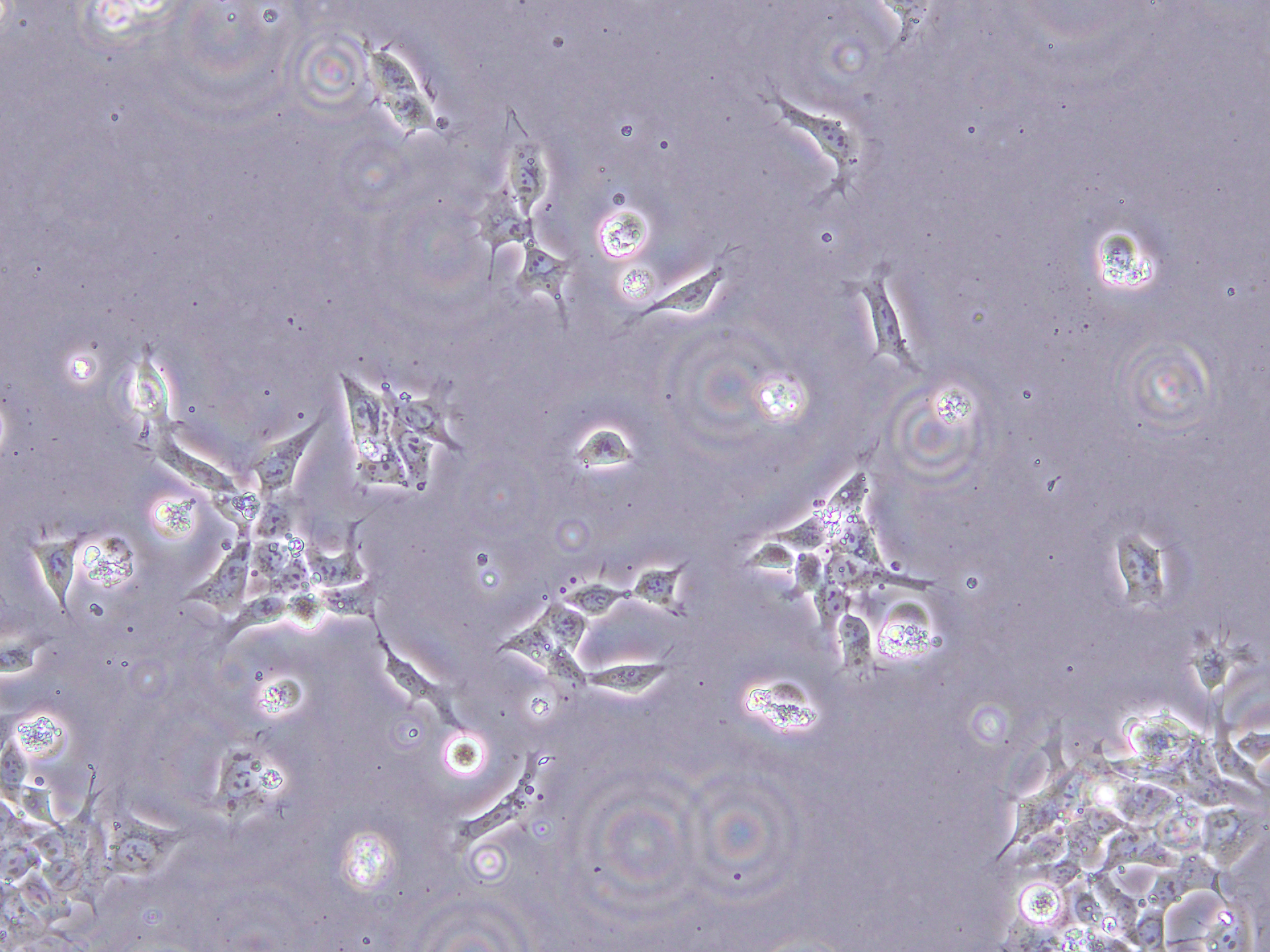

Supplement: Supplementary file 2 — Source data Fig. 1 [file 44320_2024_39_MOESM2_ESM.zip › Figure1/B/D1_20x.tif]

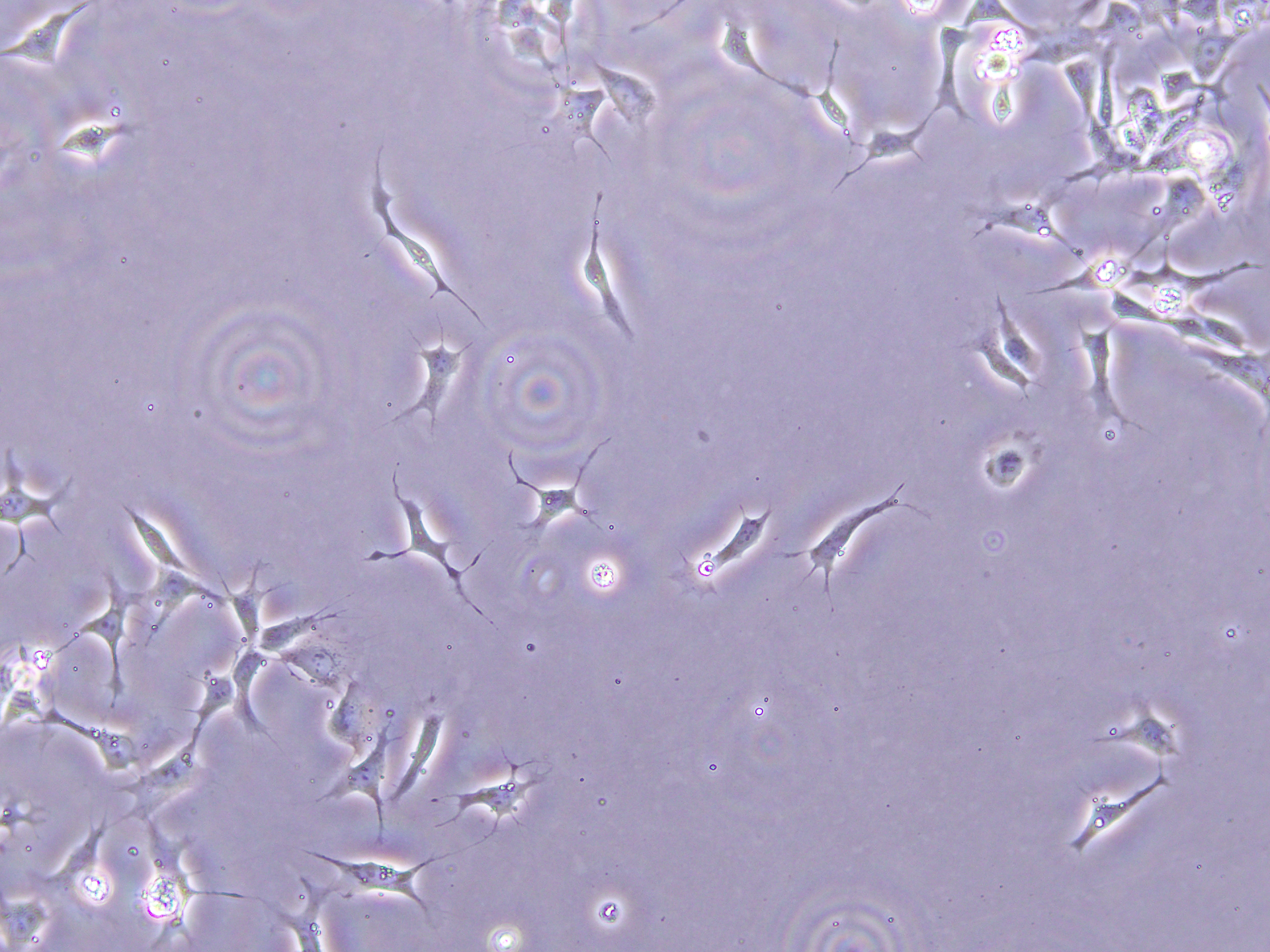

Supplement: Supplementary file 2 — Source data Fig. 1 [file 44320_2024_39_MOESM2_ESM.zip › Figure1/B/D2_20x.tif]

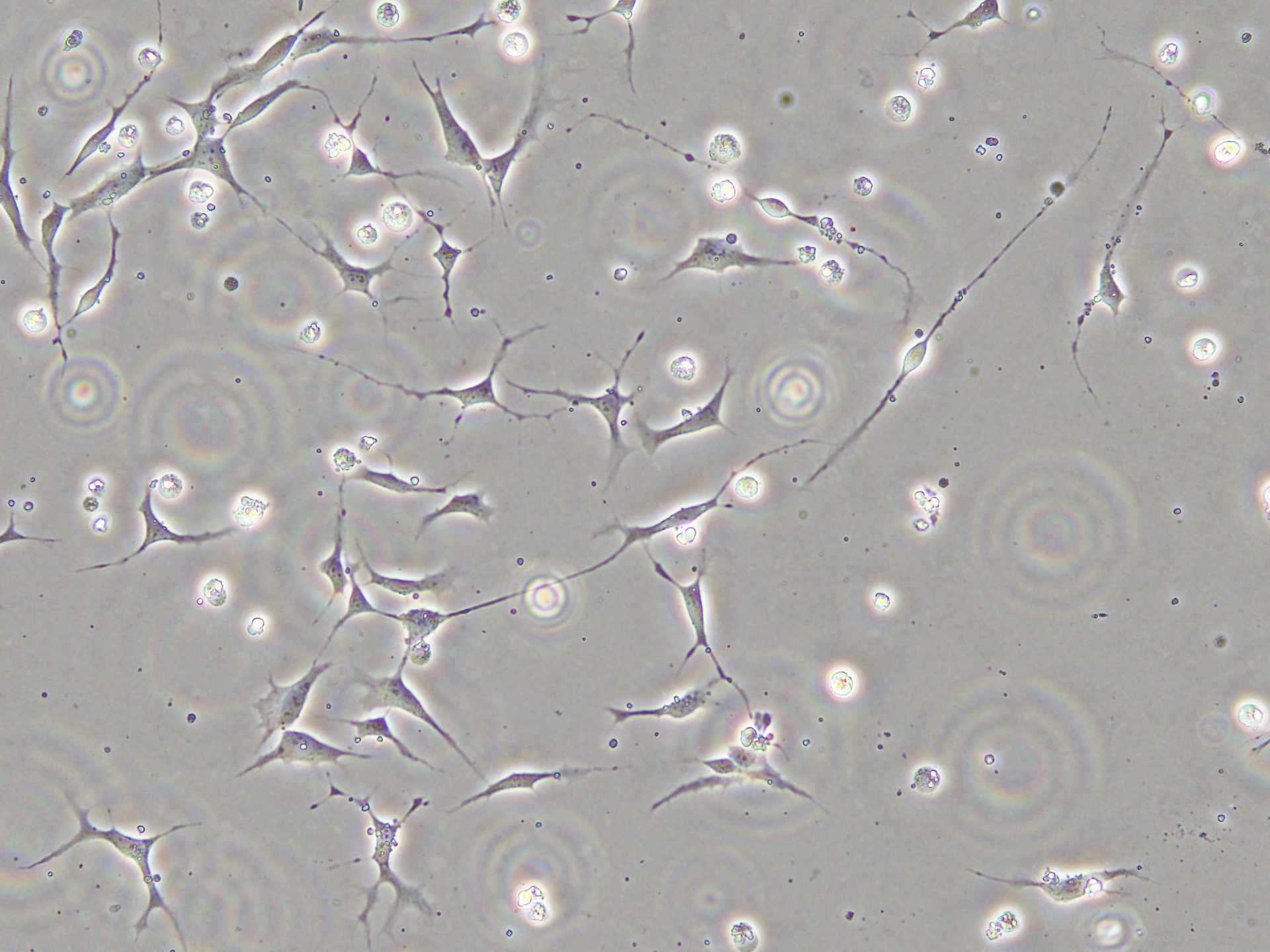

Supplement: Supplementary file 2 — Source data Fig. 1 [file 44320_2024_39_MOESM2_ESM.zip › Figure1/B/D3_20x.tif]

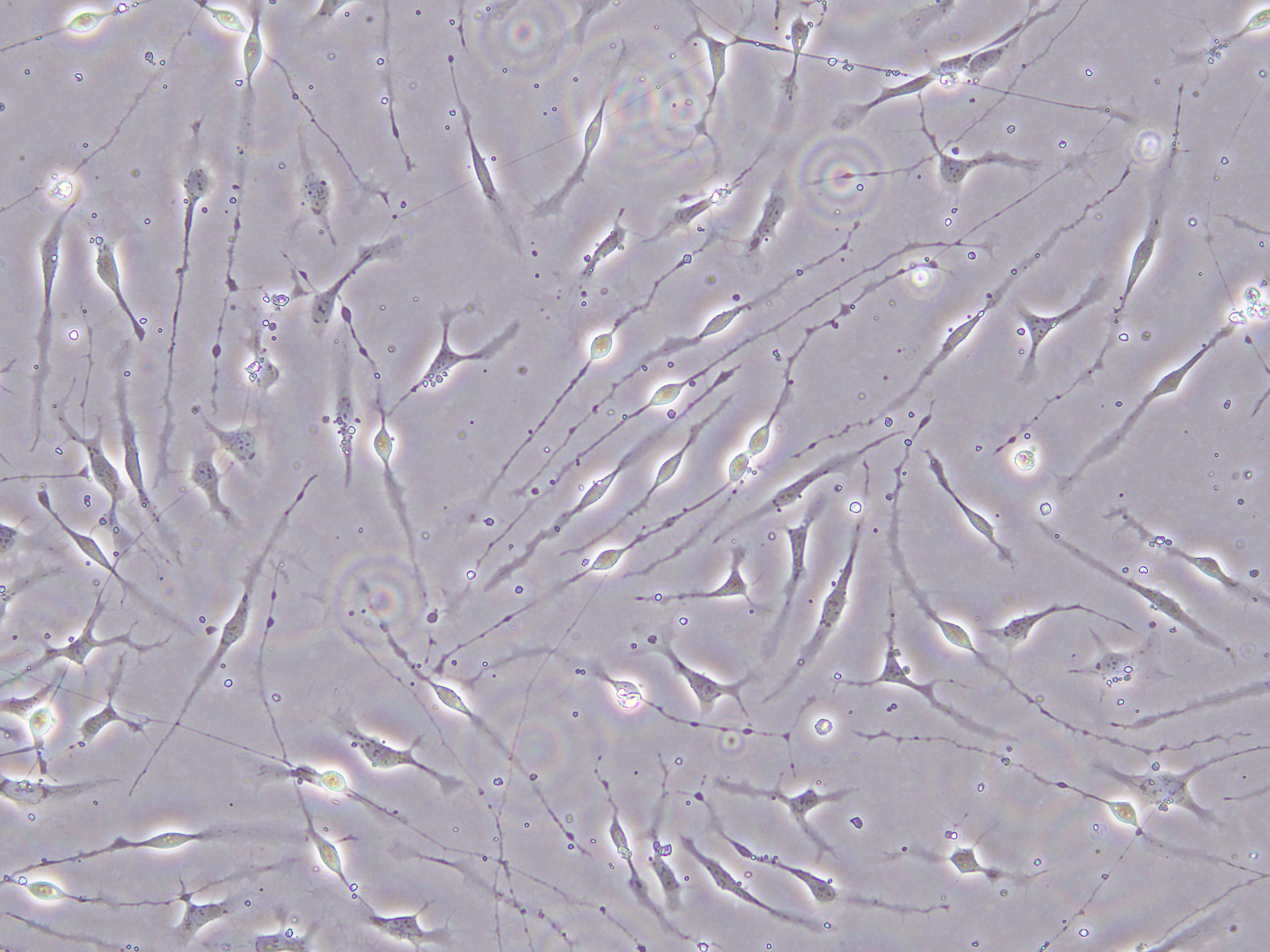

Supplement: Supplementary file 2 — Source data Fig. 1 [file 44320_2024_39_MOESM2_ESM.zip › Figure1/B/D4_20x.tif]

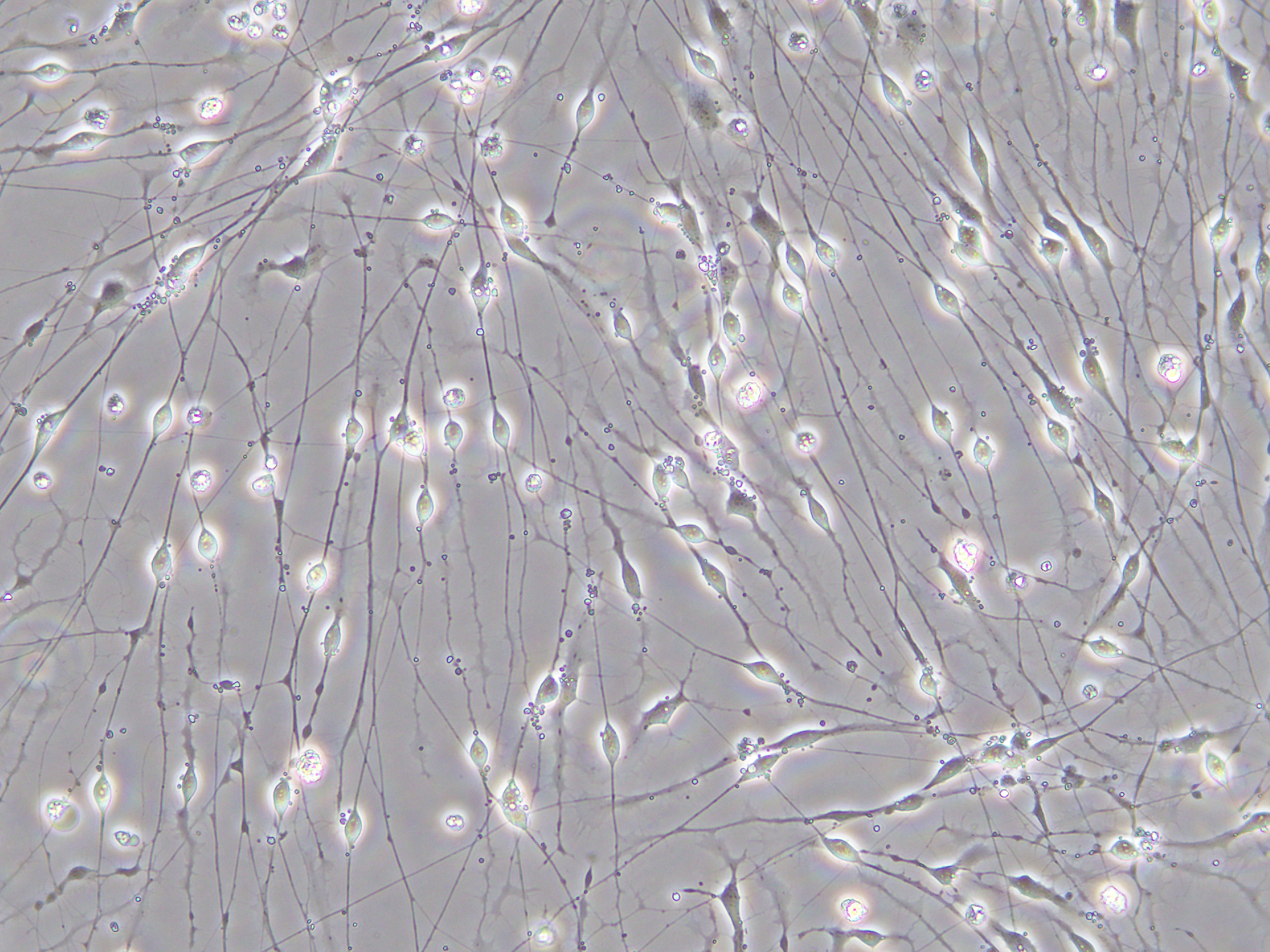

Supplement: Supplementary file 2 — Source data Fig. 1 [file 44320_2024_39_MOESM2_ESM.zip › Figure1/B/D5_20x.tif]
