# Supplementary material for: Uncovering the dynamics and consequences of RNA isoform changes during neuronal differentiation
Source: Mol Syst Biol. 2024 May 16;20(7):3. doi: 10.1038/s44320-024-00039-4 (PMC11219738; doi:10.1038/s44320-024-00039-4)
Supplement: Supplementary file 4 — Expanded View Figures [file 44320_2024_39_MOESM4_ESM.pdf]

Expanded View Figures

Figure EV1. Characterization of NGN3m neuron-like cells (related to Fig. 2).

(**A**) Z-normalized expression (transcript per million, TPM) of genes ($n = 103$) that show differential expression in at least one pairwise day-to-day comparison and that are included in the gene set 'KEGG_CELL_CYCLE' of the Molecular Signatures Database (Liberzon et al, 2015) during five days of NGN3m differentiation. The dashed vertical line indicates the transition into the post-mitotic phase. Cluster 1 ($n = 83$) and 2 ($n = 20$) include active genes in the mitotic phase (from day 0 to day 2) and the post-mitotic phase (from day 3 to day 5), respectively. (**B**) Z-normalized expression (TPM) of differentially expressed transcription factors (TFs, $n = 1094$) during five days of NGN3m differentiation. Cluster 1 ($n = 447$), 2 ($n = 268$), and 3 ($n = 379$) include TFs that were primarily expressed on day 0, from day 1 to day 3, and on day 5, respectively. (**C**) Z-normalized expression (TPM) of marker genes for different types of neurons. The neural marker genes are based on the "Neural markers guide" published by Abcam (Data ref: Abcam). All markers rely on experimental evidence. (**D**) The heatmap shows Z-normalized Spearman's correlation coefficients between day 5 NGN3m cells and the human developmental transcriptome data collection of the Developing Human Brain Atlas (Miller et al, 2014). The x-axis shows the developmental time, and the y-axis represents the brain regions. pcw: post-conception weeks; mos: months; yrs: years; MD: mediodorsal nucleus of thalamus; CBC: cerebellar cortex; S1C: primary somatosensory cortex (area S1, areas 3,1,2); IPC: posteroventral (inferior) parietal cortex; M1C: primary motor cortex (area M1, area 4); STR: striatum; V1C: primary visual cortex (striate cortex, area V1/17); A1C: primary auditory cortex (core); VFC: ventrolateral prefrontal cortex; HIP: hippocampus (hippocampal formation); ITC: inferolateral temporal cortex (area TEv, area 20); OFC: orbital frontal cortex; DFC: dorsolateral prefrontal cortex; MFC: anterior (rostral) cingulate (medial prefrontal) cortex; STC: posterior (caudal) superior temporal cortex (area 22c); and AMY: amygdaloid complex.

▶

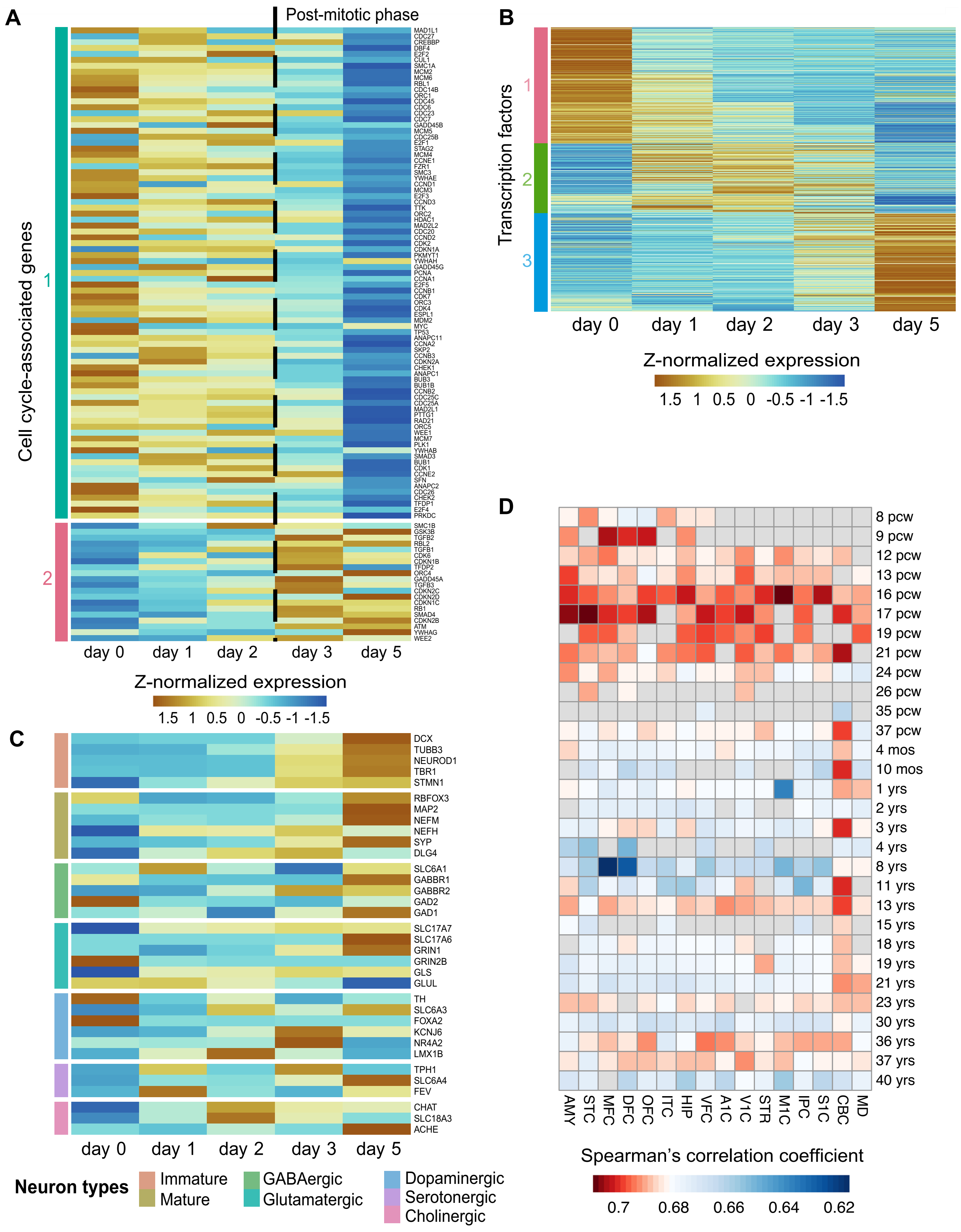
A
Post-mitotic phase
Cell cycle-associated genes
1
2
day 0 day 1 day 2 day 3 day 5
Z-normalized expression
1.5 1 0.5 0 -0.5 -1 -1.5
B
Transcription factors
1
2
3
day 0 day 1 day 2 day 3 day 5
Z-normalized expression
1.5 1 0.5 0 -0.5 -1 -1.5
C
DCX
TUBB3
NEUROD1
TBR1
STMN1
RBFOX3
MAP2
NEFM
NEFH
SYP
DLG4
SLC6A1
GABBR1
GABBR2
GAD2
GAD1
SLC17A7
SLC17A6
GRIN1
GRIN2B
GLS
GLUL
TH
SLC6A3
FOXA2
KCNJ6
NR4A2
LMX1B
TPH1
SLC6A4
FEV
CHAT
SLC18A3
ACHE
day 0 day 1 day 2 day 3 day 5
Neuron types
Immature
Mature
GABAergic
Glutamatergic
Dopaminergic
Serotonergic
Cholinergic
D
8 pcw
9 pcw
12 pcw
13 pcw
16 pcw
17 pcw
19 pcw
21 pcw
24 pcw
26 pcw
35 pcw
37 pcw
4 mos
10 mos
1 yrs
2 yrs
3 yrs
4 yrs
8 yrs
11 yrs
13 yrs
15 yrs
18 yrs
19 yrs
21 yrs
23 yrs
30 yrs
36 yrs
37 yrs
40 yrs
AMY STC MFC DFC OFC ITC HIP VFC A1C V1C STR M1C IPC S1C CBC MD
Spearman's correlation coefficient
0.7 0.68 0.66 0.64 0.62

 © The Author(s)

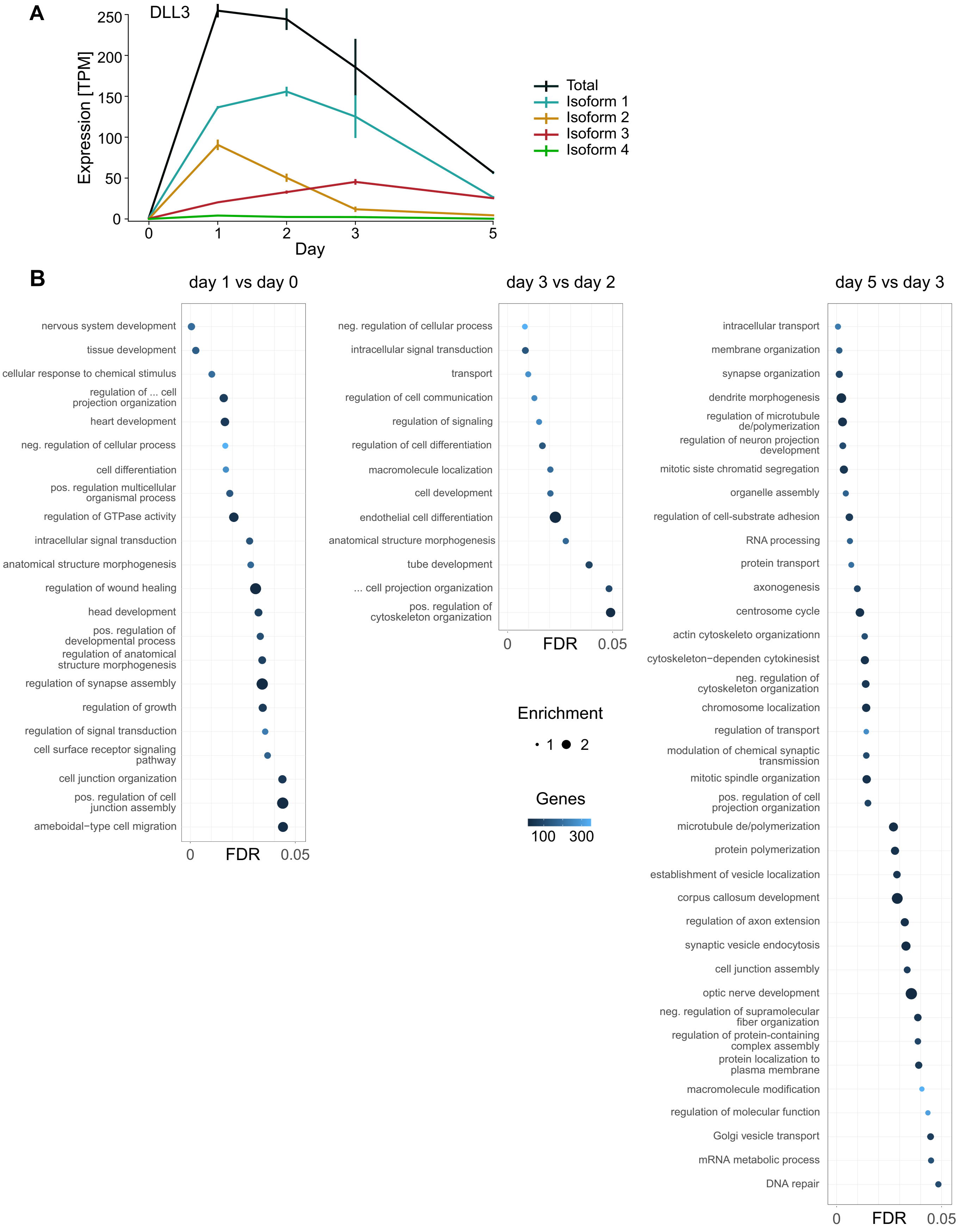
A
DLL3
Expression [TPM]
250
200
150
100
50
0
0 1 2 3 5
Day
Total
Isoform 1
Isoform 2
Isoform 3
Isoform 4
B
day 1 vs day 0
nervous system development
tissue development
cellular response to chemical stimulus
regulation of ... cell projection organization
heart development
neg. regulation of cellular process
cell differentiation
pos. regulation multicellular organismal process
regulation of GTPase activity
intracellular signal transduction
anatomical structure morphogenesis
regulation of wound healing
head development
pos. regulation of developmental process
regulation of anatomical structure morphogenesis
regulation of synapse assembly
regulation of growth
regulation of signal transduction
cell surface receptor signaling pathway
cell junction organization
pos. regulation of cell junction assembly
ameboidal−type cell migration
0 FDR 0.05
day 3 vs day 2
neg. regulation of cellular process
intracellular signal transduction
transport
regulation of cell communication
regulation of signaling
regulation of cell differentiation
macromolecule localization
cell development
endothelial cell differentiation
anatomical structure morphogenesis
tube development
... cell projection organization
pos. regulation of cytoskeleton organization
0 FDR 0.05
Enrichment
1 2
Genes
100 300
day 5 vs day 3
intracellular transport
membrane organization
synapse organization
dendrite morphogenesis
regulation of microtubule de/polymerization
regulation of neuron projection development
mitotic siste chromatid segregation
organelle assembly
regulation of cell-substrate adhesion
RNA processing
protein transport
axonogenesis
centrosome cycle
actin cytoskeleto organizationn
cytoskeleton−dependen cytokinesist
neg. regulation of cytoskeleton organization
chromosome localization
regulation of transport
modulation of chemical synaptic transmission
mitotic spindle organization
pos. regulation of cell projection organization
microtubule de/polymerization
protein polymerization
establishment of vesicle localization
corpus callosum development
regulation of axon extension
synaptic vesicle endocytosis
cell junction assembly
optic nerve development
neg. regulation of supramolecular fiber organization
regulation of protein-containing complex assembly
protein localization to plasma membrane
macromolecule modification
regulation of molecular function
Golgi vesicle transport
mRNA metabolic process
DNA repair
0 FDR 0.05

© The Author(s)

Figure EV2. Significantly enriched gene ontology (GO) terms for genes with differential isoform expression patterns during NGN3m differentiation (related to Fig. 3).

(**A**) Quantification of total gene expression of *DLL3* and relative abundance of four transcript isoforms inferred from RNA-seq time-course data. Expression levels were normalized as transcript per million (TPM). (**B**) All significant GO terms (Fisher's exact test, FDR < 0.05, level 0) from genes with differential isoform expressions between indicated days of the differentiation course.

© The Author(s)

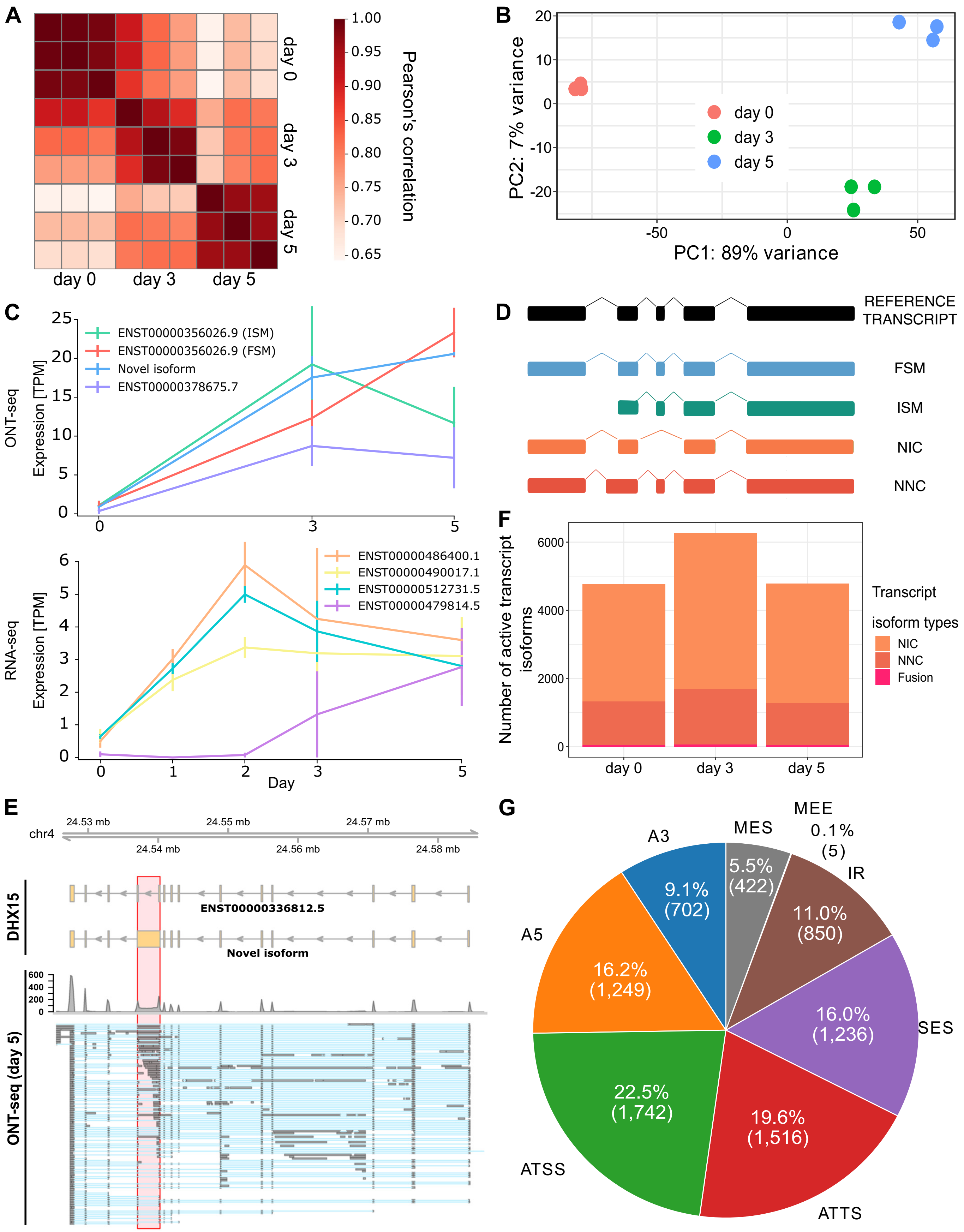
A
day 0
day 3
day 5
Pearson's correlation
1.00
0.95
0.90
0.85
0.80
0.75
0.70
0.65
B
PC1: 89% variance
PC2: 7% variance
day 0
day 3
day 5
C
ONT-seq
Expression [TPM]
ENST00000356026.9 (ISM)
ENST00000356026.9 (FSM)
Novel isoform
ENST00000378675.7
RNA-seq
ENST00000486400.1
ENST00000490017.1
ENST00000512731.5
ENST00000479814.5
Day
D
REFERENCE TRANSCRIPT
FSM
ISM
NIC
NNC
F
Number of active transcript isoforms
Transcript isoform types
NIC
NNC
Fusion
E
chr4
24.53 mb
24.55 mb
24.57 mb
24.54 mb
24.56 mb
24.58 mb
DHX15
ENST00000336812.5
Novel isoform
ONT-seq (day 5)
G
MEE 0.1% (5)
MES 5.5% (422)
A3 9.1% (702)
IR 11.0% (850)
A5 16.2% (1,249)
SES 16.0% (1,236)
ATSS 22.5% (1,742)
ATTS 19.6% (1,516)

◀ **Figure EV3. High reproducibility of ONT-seq replicate datasets and identification of new RNA isoforms during NGN3m differentiation (related to Fig. 4).**

(**A**) Pearson's correlation analysis of gene expression levels (transcript per million, TPM) of ONT-seq replicate datasets for the different time points. (**B**) PCA plot of expressed genes throughout the differentiation course as measured by ONT-seq. (**C**) RNA isoform expression profiles for *MMP23B* directly observed by ONT-seq (top panel) or inferred from RNA-seq data (lower panel). TPM: transcripts per kilobase million. ISM: Incomplete Splice Match. FSM: Full Splice Match. (**D**) Schematic view of main types of transcript isoforms that were identified from ONT-seq data and classified based on their agreement with the intron structure of the reference transcript by SQANTI3. FSM: matches all splice junctions (SJs) perfectly; ISM: matches the reference SJs partially; NIC: novel isoform with a new combination of known splice sites; and NNC: novel isoform with at least a new splicing site. (**E**) ONT-seq identifies a novel RNA isoform for *DHX15* that is expressed at day 5 of NGN3m differentiation. A red box highlights the differing region. (**F**) Number of new isoforms that show active expression (mean TPM > 5 across replicates) at day 0 (NIC $n = 3456$, NNC $n = 1275$, Fusion $n = 44$), day 3 (NIC $n = 4584$, NNC $n = 1617$, Fusion $n = 66$), and day 5 (NIC $n = 3518$, NNC $n = 1219$, Fusion $n = 53$) of NGN3m differentiation as measured by ONT-seq. (**G**) Fractions of alternative transcription and splicing mechanisms of differentially expressed isoforms, as measured by ONT-seq, across all consecutive day-to-day comparisons.

 © The Author(s)

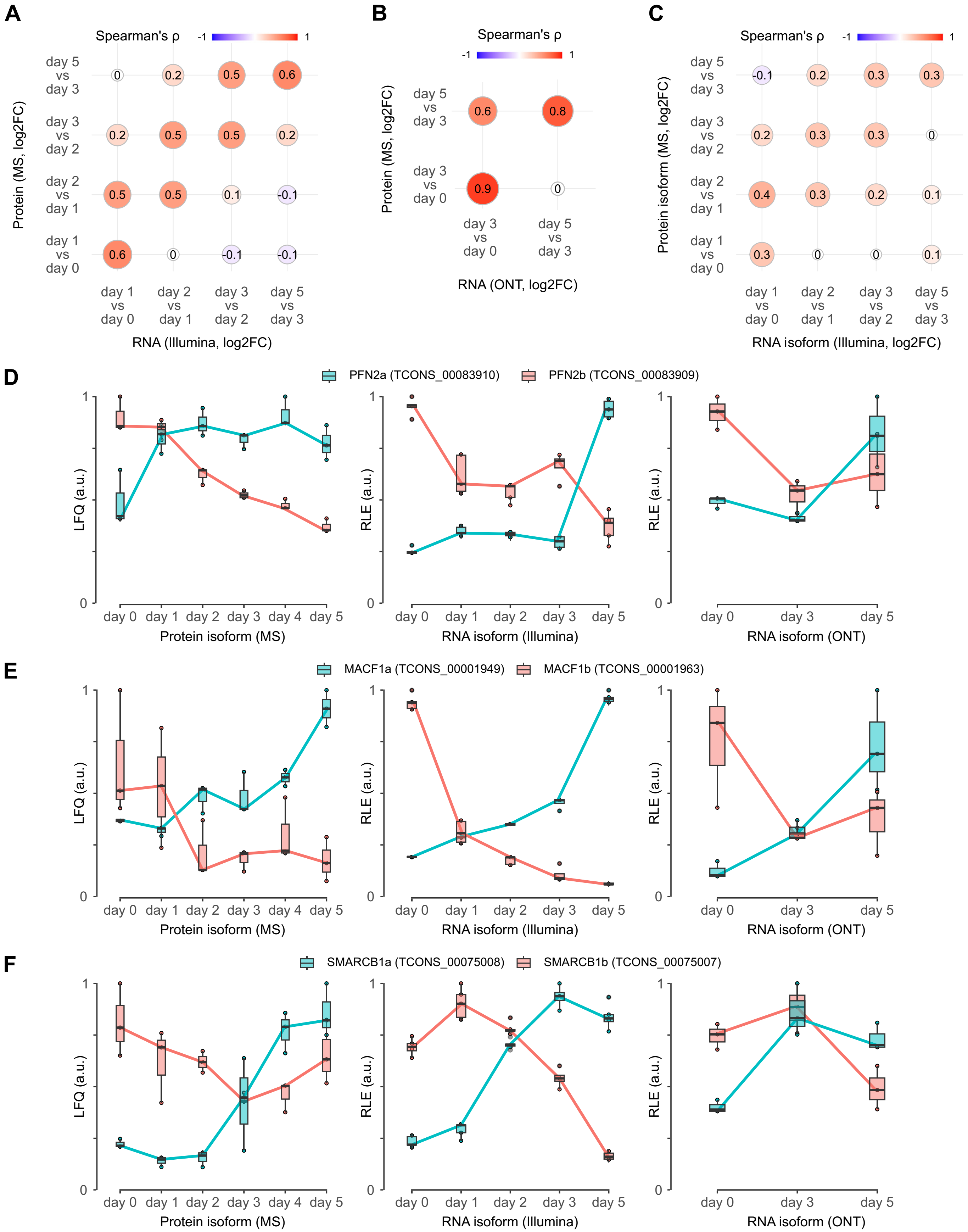
A
Spearman's ρ
Protein (MS, log2FC)
RNA (Illumina, log2FC)
B
Spearman's ρ
Protein (MS, log2FC)
RNA (ONT, log2FC)
C
Spearman's ρ
Protein isoform (MS, log2FC)
RNA isoform (Illumina, log2FC)
D
PFN2a (TCONS_00083910)
PFN2b (TCONS_00083909)
LFQ (a.u.)
RLE (a.u.)
Protein isoform (MS)
RNA isoform (Illumina)
RNA isoform (ONT)
E
MACF1a (TCONS_00001949)
MACF1b (TCONS_00001963)
F
SMARCB1a (TCONS_00075008)
SMARCB1b (TCONS_00075007)

Figure EV4. Correlation of proteomics and transcriptomics data (related to Fig. 5).

(**A**) Spearman's correlation coefficient of log2 fold changes of RNA- and protein-levels between indicated days for all expressed genes. RNA levels were measured by RNA-seq and proteins by mass spectrometry (MS). Correlation analysis was restricted to genes and proteins that were detected in at least one time point. (**B**) Spearman's correlation coefficient of log2 fold changes of RNA- and protein-levels between indicated days for differentially expressed genes (RNA: padj <0.05 and |log2FC| > 0.59, Wald test; proteins: padj <0.1 and |log2FC| > 0.32, moderated t-test). RNA levels were measured by ONT-seq and proteins by mass spectrometry (MS). Correlation analysis was restricted to genes and proteins that were differentially expressed between at least one time point. (**C**) Spearman's correlation coefficient of log2 fold changes of RNA and protein isoforms across consecutive days. Logarithmic fold changes were measured by RNA-seq and mass spectrometry (MS). Correlation analysis was restricted to protein isoforms competing with at least one other protein isoform from the same gene ($n = 272$). (**D–F**) Depicted are protein and RNA isoform quantifications for PFN2 (**D**), MACF1 (**E**), and SMARCB1 (**F**) across the differentiation days. The boxplots show protein isoform quantifications (LFQ intensities, normalized to 1) from mass spectrometry (MS, triplicates) (left) and RNA quantification (relative log expression (RLE), normalized to 1) from Illumina (four to five replicates) and ONT (triplicates) sequencing data (right). The median values are depicted as the center. The box is defined by the first to the third interquartile range. The whiskers extend this interquartile range by a factor of 1.5, not exceeding the minimum or maximum values. Biological replicate measurements are depicted as individual dots.

© The Author(s)

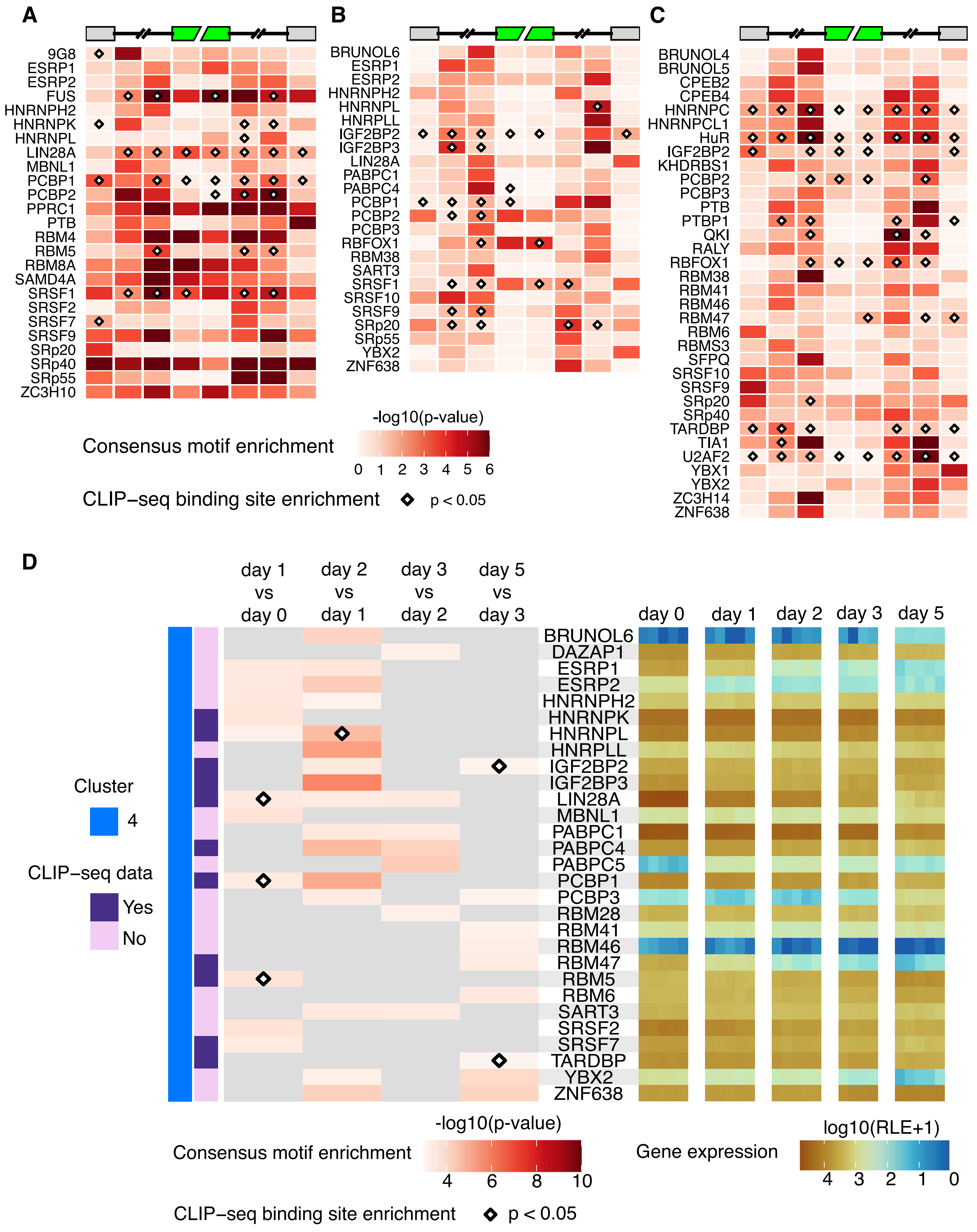
A
9G8
ESRP1
ESRP2
FUS
HNRNPH2
HNRNPK
HNRNPL
LIN28A
MBNL1
PCBP1
PCBP2
PPRC1
PTB
RBM4
RBM5
RBM8A
SAMD4A
SRSF1
SRSF2
SRSF7
SRSF9
SRp20
SRp40
SRp55
ZC3H10
B
BRUNOL6
ESRP1
ESRP2
HNRNPH2
HNRNPL
HNRPLL
IGF2BP2
IGF2BP3
LIN28A
PABPC1
PABPC4
PCBP1
PCBP2
PCBP3
RBFOX1
RBM38
SART3
SRSF1
SRSF10
SRSF9
SRp20
SRp55
YBX2
ZNF638
C
BRUNOL4
BRUNOL5
CPEB2
CPEB4
HNRNPC
HNRNPCL1
HuR
IGF2BP2
KHDRBS1
PCBP2
PCBP3
PTB
PTBP1
QKI
RALY
RBFOX1
RBM38
RBM41
RBM46
RBM47
RBM6
RBMS3
SFPQ
SRSF10
SRSF9
SRp20
SRp40
TARDBP
TIA1
U2AF2
YBX1
YBX2
ZC3H14
ZNF638
-log10(p-value)
Consensus motif enrichment
0 1 2 3 4 5 6
CLIP–seq binding site enrichment
p < 0.05
D
day 1 vs day 0
day 2 vs day 1
day 3 vs day 2
day 5 vs day 3
day 0
day 1
day 2
day 3
day 5
BRUNOL6
DAZAP1
ESRP1
ESRP2
HNRNPH2
HNRNPK
HNRNPL
HNRPLL
IGF2BP2
IGF2BP3
LIN28A
MBNL1
PABPC1
PABPC4
PABPC5
PCBP1
PCBP3
RBM28
RBM41
RBM46
RBM47
RBM5
RBM6
SART3
SRSF2
SRSF7
TARDBP
YBX2
ZNF638
Cluster
4
CLIP–seq data
Yes
No
-log10(p-value)
Consensus motif enrichment
4 6 8 10
Gene expression
log10(RLE+1)
4 3 2 1 0
CLIP–seq binding site enrichment
p < 0.05

Figure EV5. Differential RNA binding patterns during NGN3m differentiation (related to Fig. 6).

(**A–C**) Heatmap illustrating the spatial enrichment patterns of RBP consensus motifs in the vicinity of differentially included exons between (**A**) day 0 and day 1, (**B**) day 1 and day 2, and (**C**) day 3 and day 5 of NGN3m neurogenesis as determined by scanning motifs in adjacent sequence segments. A schematic view of sequence segments scanned by rMAPS2 for the occurrences of consensus motifs in exon skipping events is shown above the respective heatmap. Alternatively regulated exons are depicted as a green box. Only RBPs with a motif enrichment *p*-value (Wilcoxon's rank sum test) <0.001 are shown (related to Fig. 6C). The diamond-shaped labels indicate regions with enriched RBP binding sites (*p*-value < 0.05, Fisher's exact test) from CLIP-seq datasets (Data ref: Zhao et al, 2021). (**D**) Left: Heatmap depicting the temporal enrichment patterns of potential binding sites of class 4 RBPs relative to differentially included exons during the differentiation course (related to Fig. 6E). For each RBP and day-to-day comparison, the minimum enrichment *p*-value across different regions is shown. The diamond-shaped labels highlight the co-occurrence of enriched RBP binding sites (*p*-value < 0.05, Fisher's exact test) and enriched consensus motifs (*p*-value < 0.001, Wilcoxon's rank sum test) in the same region (Data ref: Zhao et al, 2021). Right: Quantification of total gene expression for class 4 RBPs from RNA-seq time-course data. Expression levels were normalized as relative-log expression (RLE) and log10 transformed.

 © The Author(s)
